# Large-scale network interactions supporting item-context memory formation

**DOI:** 10.1371/journal.pone.0210167

**Published:** 2019-01-10

**Authors:** Sungshin Kim, Joel L. Voss

**Affiliations:** 1 Center for Neuroscience Imaging Research, Institute for Basic Science (IBS), Suwon, Republic of Korea; 2 Sungkyunkwan University (SKKU), Suwon, Republic of Korea; 3 Department of Medical Social Sciences, Ken and Ruth Davee Department of Neurology, Department of Psychiatry and Behavioral Sciences, Feinberg School of Medicine, Northwestern University, Chicago, United States of America; University of Cambridge, UNITED KINGDOM

## Abstract

Episodic memory is thought to involve functional interactions of large-scale brain networks that dynamically reconfigure depending on task demands. Although the hippocampus and closely related structures have been implicated, little is known regarding how large-scale and distributed networks support different memory formation demands. We investigated patterns of interactions among distributed networks while human individuals formed item-context memories for two stimulus categories. Subjects studied object-scene and object-location associations in different fMRI sessions. Stimulus-responsive brain regions were organized based on their fMRI interconnectivity into networks and modules using probabilistic module-detection algorithms to maximize measurement of individual differences in modular structure. Although there was a great deal of consistency in the modular structure between object-scene and object-location memory formation, there were also significant differences. Interactions among functional modules predicted later memory accuracy, explaining substantial portions of variability in memory formation success. Increased interactivity of modules associated with internal thought and anti-correlation of these modules with those related to stimulus-evoked processing robustly predicted object-scene memory, whereas decreased interactivity of stimulus-evoked processing modules predicted object-location memory. Assessment of individual differences in network organization therefore allowed identification of distinct patterns of functional interactions that robustly predicted memory formation. This highlights large-scale brain network interactions for memory formation and indicates that although networks are largely robust to task demands, reconfiguration nonetheless occurs to support distinct memory formation demands.

## Introduction

Cognition is supported by large-scale brain networks [1] that can be studied using connectivity analyses of MRI and fMRI data [2]. The critical role of hippocampus in episodic memory has been established, and fMRI connectivity analyses have implicated a broader network of regions that are thought to interact closely with the hippocampus [3–5]. Consistent with this fMRI evidence for a broader episodic memory network, lesions of regions such as prefrontal and parietal cortex disrupt some measures of episodic memory [6, 7]. Many experiments have focused on broad networks of the hippocampus supporting associative memory for stimulus pairings, such as the relationship between an item and the context in which it was presented (item-context memory) [3]. Item-context memory has been associated with interactivity of the hippocampus with a variety of cortical regions forming anterior-temporal and posterior-medial networks [3–5].

However, fMRI studies of the network-basis of memory have mainly used targeted analyses that focus either exclusively on the hippocampal formation and its connectivity [8–10] or only consider connectivity in relation to specific components of task-related signal [6, 11, 12]. In other domains, such as language, broader interactions have been explored, thus providing information on how the structure of large-scale brain networks vary with different cognitive task demands [13–15]. Although some studies have focused on network interactions supporting episodic memory retrieval [16–18], such interactions during memory formation have rarely been explored.

Here, we sought to evaluate the potential for fMRI analyses of large-scale networks during memory formation by identifying relevant interactions that support distinct types of item-context memory formation. Item-context memory is a broad category that includes a variety of different stimulus and task formats [3, 5, 19]. Little is known regarding variation in brain networks that support different types of item-context memory. Although evidence taken from many rodent and human studies suggests specialization along the hippocampal long axis and its associated networks based on the nature of stimuli comprising item-context associations [19], we are unaware of within-experiment comparisons to identify distinct networks for item-context associations of different formats. We developed tasks involving episodic memory for two types of item-context associations: object-scene associations and object-location associations. In the analysis of networks associated with the two memory tasks, we used a relatively novel application of probabilistic connectivity considering inter-subject variability in functional networks [13, 20]. This method provides estimates of inter-subject variability in network structure, thus yielding greater potential for identification of connectivity patterns accounting for inter-subject variability in memory performance. This allowed us to identify functional network interactions associated with memory formation in different ways for two standard item-context memory formats.

## Materials and methods

### Participants

Data were collected from 30 subjects (21 females and 9 males; mean age, 25.6 years; age range = 18–34 years). All participants had normal or corrected-to-normal vision and did not report any neurological disorder or current drug use. They were eligible based on standard MRI safety screening. Data from 17 of the subjects were collected as part of the baseline assessment for a two-week noninvasive brain stimulation experiment, and effects of brain stimulation were reported elsewhere [21]. All participants provided written informed consent and were compensated for their participation. The experiments were conducted according to the Declaration of Helsinki and approved by Northwestern University Institutional Review Board.

### Task procedures and experiment design

**Fig 1:**
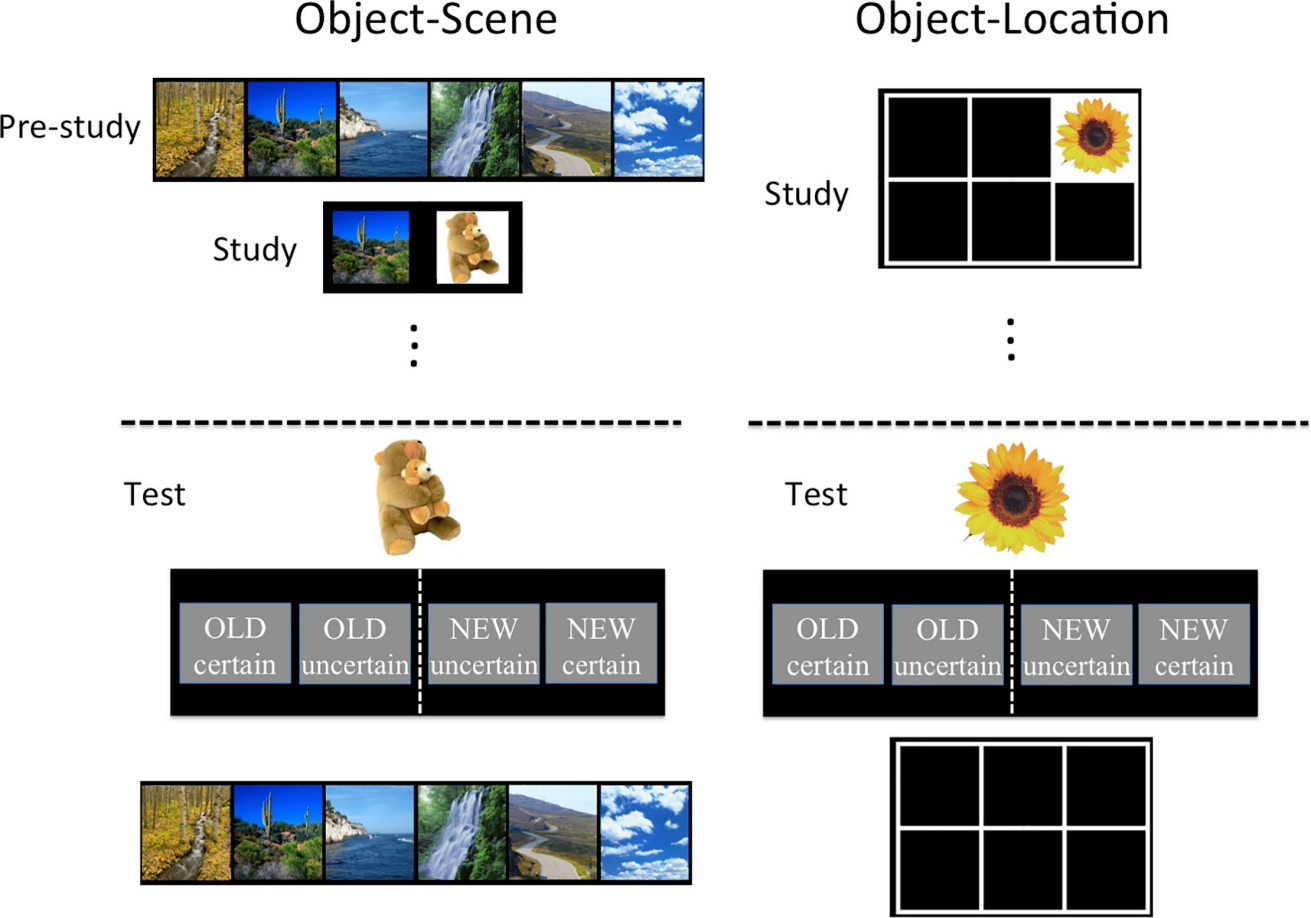
Object-scene and object-location memory tasks. In the object-scene task, subjects were first pre-familiarized with six scenes. During the study phase, subjects studied 36 trial-unique objects, each paired with one scene (six objects were paired with each scene) in randomized order. Three additional non-tested items were used as primacy and recency buffers. During the corresponding test, old objects were presented intermixed with an equal number of new objects. Subjects made old/new recognition judgments mixed with a confidence judgment. For all old items, subjects then selected the scene that was paired with the object. The object-location task followed a similar format, except that there were six possible screen locations instead of six possible scene associates. Object and scene images were respectively taken from publically available sources, which were described in Brady et al.[22] and Hannula et al.[23], with no copyright protection via the internet for display purposes.

fMRI scanning occurred during two item-context associative memory tasks of different formats: object-scene and object-location (Fig 1). These tasks differed in the type of contextual information for which memory was tested, but were matched in many other parameters, including the same number of contexts paired with each item, similar potential for contextual interference across trials, and difficulty. In the object-scene task, 36 trial-unique objects were arbitrarily paired with one of six scene images, whereas in the object-location task, 36 trial-unique objects were arbitrarily paired with one of six screen locations. Each context (scene or location) was paired with exactly six objects. The images for trial-unique objects and scene objects were respectively taken from stimulus sets developed for research purposes, described in Brady et al.[22] (Computational Visual Cognition Lab, http://cvcl.mit.edu/MM, Cambridge, MA) and Hannula et al.[23]. There were five different sets of object images each with six corresponding scene images that were generated via random selection from the overall set, and these five sets were counterbalanced across subjects. Within each set, images of objects were randomly assigned to condition (old versus new), as described below.

For the object-scene task, subjects were familiarized with the six scenes prior to the study phase (scenes were viewed freely and subjects provided verbal labels aloud for each). During the object-scene study session, each object was presented next to its corresponding scene for 1.5 seconds, followed by random inter-stimulus interval ranging 2 to 6 seconds (mean = 4 s). An additional six object-scene pairs were presented, three at the beginning of the study session and three at the end, but these were not later tested, to reduce primacy and recency effects [24]. The corresponding object-scene memory test followed the study session by a delay of approximately 2 minutes during which subjects were reminded of instructions. Seventy-two objects were presented, half of which were old (presented during the study session) and the other half new, in randomized order. On each trial, the object appeared first for 2 s, followed by a random delay of 2–6 seconds (mean = 4 s) with a white fixation cross at the center of the screen. Then a prompt appeared asking subjects to categorize the object as old or new, each with two levels of confidence (“certain” or “uncertain”). Subjects had three seconds to make the response and trials without any response were considered “missed” and excluded in further analysis, with fewer than one missed trial on average for both tasks (object-scene: 0.77 ± 0.35 trials, object-location: 0.40 ± 0.16 trials, mean ± SE). For old objects only, subjects then were asked to respond to indicate the corresponding studied scene context. This occurred for all old objects, irrespective of whether the old/new response was correct. Immediately after subjects’ old/new response or three seconds after the prompt appeared without subjects’ response, the six possible scene context images were shown, and the subject had to select the scene context paired with the object. This response portion lasted five seconds and the trials without responses were counted as incorrect. An inter-trial interval of 2–6 seconds (mean = 4 s) separated the response period from the next trial.

The object-location memory task was identical to the object-scene memory task, except for the nature of the context. Instead of being presented paired with one of six scenes, trial-unique objects during study were presented at one of six possible screen locations, and memory for these locations was probed in the corresponding test session using the same format as in the object-scene task (Fig 1).

The study and test portions of the object-scene and object-location memory tasks were presented consecutively, with the order of tasks counterbalanced across subjects. Subjects were familiarized with the overall procedure before the experimental session and therefore were aware of the upcoming memory tests during the study phases of the tasks. The tasks were performed while subjects laid supine in the scanner, viewing an MRI-compatible LCD monitor via a mirror attached to the head coil. Objects and scene images subtended approximately 3×3 degrees of visual angle. The 2×3 grid used for the object-location task subtended approximately 6x9 degrees of visual angle. Responses during the memory tests were registered using an MRI-compatible mouse, which rested on a rigid plastic surface and was controlled with the right hand.

### MRI data collection and preprocessing

MRI data were collected during the study and test sessions of each task, but only study sessions are analyzed here as our focus is on memory formation rather than retrieval. MRI data were collected using two Siemens 3T TIM Prisma whole-body scanners with 64-channel head/neck coils. A structural image was acquired before the task to provide anatomical location (MP-RAGE T_1_-weighted scans; voxel size: 1mm^3^; field of view: 256 mm, 151 sagittal slices). Whole-brain functional images were acquired during the tasks, with a 2000-ms repetition time, 20-ms echo time, 210 mm field of view, 80° flip angle, 1.7×1.7×1.7 mm isotropic voxels, and multi band factor of 2. Preprocessing of fMRI data used AFNI software version 4.56 [25]. The procedure includes motion correction with estimating six rigid body motions (3dvolreg), correcting multi-band slice-timing (3dTshift), coregistration of skull stripped structural image (3dSkullStrip) and functional images with using a local Pearson correlation as a cost function (align_epi_anat.py), normalization to stereotactic space using a standard template (@auto_tlrc), which is manually Talairched version of the Colin_N27 dataset (TT_N27 in AFNI), and spatial smoothing with a 3-mm FWHM Gaussian kernel (3dmerge). The smoothed fMRI time course data were band-pass filtered with 0.01–0.1 Hz and de-spiked (3dBandpass). The duration of each scan varied slightly based on ISI randomization, varying from 115 to 132 volumes for the scene task and from 117 to 133 volumes for the object-location task. There was no significant difference in duration between tasks (t(29) = 0.57, P = 0.57).

### Region of interest selection

We defined functional-anatomical ROIs via a two-step procedure. First, we identified regions that were task-responsive, as defined by univariate analysis of stimulus-evoked activity pooled for both object-scene and object-location tasks. For each subject, the estimated BOLD response to all stimuli was obtained via a general linear model incorporating hemodynamic response deconvolution (3dDeconvolve). Regressors of interest included all stimulus onsets for the object-scene and object-location tasks and six parameters for rigid-body estimates were entered as regressors of no interest. The hemodynamic response was modeled as six tent functions from 0 sec to 10 sec after stimulus onsets with peaks every 2 seconds (aligned to the TR). Group-level one-sample t-test (3dttest++) was used to identify all task-responsive voxels, whose sum of 12 beta coefficients of tent functions (six from each of object-scene and object-location regressors) were significantly different from zero with a lenient threshold, P < 0.05 uncorrected, 2-tailed. We used this lenient threshold for network construction and thus make no claims about statistical significance of the activation map. Also, note that univariate modeling was used for ROI definition, but was not otherwise part of the connectivity analysis (see below). Voxels, whose evoked activities by stimuli were significantly different from zero, were labeled according to whether they showed positive evoked responses (task-positive) versus negative evoked responses (task-negative)[14, 26–28] (see S1 Fig). We then partitioned these functional activation maps according to structural atlases. We considered 85 anatomical regions of interest (ROIs), with 70 cortical ROIs taken from the Desikan-Killiany atlas and 15 subcortical ROIs taken from the FreeSurfer atlas provided in AFNI [29, 30]. These 85 ROIs are listed in S1 Table. The task-positive and task-negative voxels defined via the univariate fMRI analysis were inclusively masked by these anatomical ROIs and each surviving voxel was labeled for membership to one of each of the 85 anatomical ROIs. For each of the anatomical ROIs having greater than 10 surviving task-positive or task-negative voxels, we formed a functional-anatomical ROI out of all the task-negative or task-positive voxels. No functional-anatomical ROIs resulted for 7 of the 85 anatomical ROIs. Both task-positive and task-negative functional-anatomical ROIs resulted for 40 of the 85 anatomical ROIs. Either task-positive or task-negative functional-anatomical ROIs resulted for the remaining 38 anatomical ROIs. Overall, this resulted in 118 functional-anatomical ROIs, with 53 task-positive ROIs and 65 task-negative ROIs (Tables 1 and 2 and S2 Table).

**Table 1:** Labels of ROIs in the task-positive network.

Object-Scene task		Object-Location task	
Dorsal/Ventral visual module (POS1)	**R-lateral orbitofrontal**	Dorsal/Ventral visual module (POS1)	
	L-cuneus		L-cuneus
	R:L-cerebellar cortex		R:L-cerebellar cortex
	R:L-fusiform		R:L-fusiform
	R:L-inferior parietal		R:L-inferior parietal
	R:L-inferior temporal		R:L-inferior temporal
	R:L-lateral occipital		R:L-lateral occipital
	R:L-lingual		R:L-lingual
	R:L-precuneus		R:L-precuneus
	R:L-superior parietal		R:L-superior parietal
Fronto-parietal-limbic module (POS2)	L-middle temporal	Fronto-parietal module (POS2)	L-middle temporal
	L-pars orbitalis		L-pars opercularis
	L-postcentral		L-postcentral
	L-superior temporal sulcus		L-superior temporal sulcus
	L-supramarginal		L-supramarginal
	R:L-caudal middle frontal		R:L-caudal middle frontal
	R:L-pars triangularis		R:L-pars triangularis
	R:L-precentral		R:L-precentral
	R:L-rostral middle frontal		R:L-rostral middle frontal
	R:L-superior frontal		R:L-superior frontal
	R-caudal anterior cingulate	Fronto-limbic (POS3)	R-caudal anterior cingulate
	R-entorhinal		R-entorhinal
	L-amygdala		L-amygdala
	L-pallidum		L-pallidum
	**L-pars opercularis**		**L-pars orbitalis**
	R:L-caudate		R:L-caudate
	R:L-dosal thalamus		R:L-dorsal thalamus
	R:L-hippocampus		R:L-hippocampus
	R:L-insula		R:L-insula
	L-lateral orbitofrontal		R:L-lateral orbitofrontal
	R:L-parahippocampal		R:L-parahippocampal
	R:L-putamen		R:L-putamen
	R:L-ventral diencephalon		R:L-ventral diencephalon

Note: Bold indicates ROIs of which modules are assigned differently between object-scene and object-location tasks. L–Left hemisphere, R–Right hemisphere

**Table 2:** Labels of ROIs in the task-negative network.

Object-Scene task		Object-Location task	
Default-extended-limbic module (NEG1)	R-frontal pole	Default-extended module (NEG1)	R-frontal pole
	R-inferior temporal		R-inferior temporal
	R-pars orbitalis		R-pars orbitalis
	R-superior temporal sulcus		R-superior temporal sulcus
	R:L-caudal anterior cingulate		R:L-caudal anterior cingulate
	R:L-caudal middle frontal		R:L-caudal middle frontal
	R:L-inferior parietal		R:L-inferior parietal
	R:L-isthmus cingulate		R:L-isthmus cingulate
	R:L-lateral orbitofrontal		R:L-lateral orbitofrontal
	R:L-medial orbitofrontal		R:L-medial orbitofrontal
	R:L-middle temporal		R:L-middle temporal
	R:L-posterior cingulate		R:L-posterior cingulate
	R:L-precuneus		R:L-precuneus
	R:L-rostral anterior cingulate		R:L-rostral anterior cingulate
	R:L-rostral middle frontal		R:L-rostral middle frontal
	R:L-superior frontal		R:L-superior frontal
			**L-hippocampus**
	R-hippocampus	Default-limbic module (NEG3)	R-hippocampus
	R-temporal pole		R-temporal pole
	R-dorsal thalamus		R-dorsal thalamus
	**R-putamen**		**R:L-putamen**
	R:L-accumbens		R:L-accumbens
	R:L-caudate		R:L-caudate
			**R:L-ventral diencephalon**
Transitional module (NEG2)	**R:L-ventral diencephalon**	Transitional module (NEG2)	* *
	R-pars triangularis		R-pars triangularis
	L-dorsal thalamus		L-dorsal thalamus
	**L-hippocampus**		
	L-transverse temporal		L-transverse temporal
	R:L-cuneus		R:L-cuneus
	R:L-lingual		R:L-lingual
	R:L-paracentral		R:L-paracentral
	R:L-pars opercularis		R:L-pars opercularis
	R:L-postcentral		R:L-postcentral
	R:L-precentral		R:L-precentral
	R:L-superior parietal		R:L-superior parietal
	R:L-superior temporal		R:L-superior temporal
	R:L-supramarginal		R:L-supramarginal
	R:L-insula		R:L-insula
	R:L-cerebellar cortex		R:L-cerebellar cortex
	**L-putamen**		* *

Note: Bold indicates ROIs of which modules are assigned differently between object-scene and object-location tasks. L–Left hemisphere, R–Right hemisphere

### Probabilistic fMRI connectivity analysis

For each of the memory tasks, we averaged the preprocessed fMRI time series spatially within each of 118 functional-anatomical ROIs and cross-correlated between all the pairs of the averaged signals, separately for each subject. The resulting constructed connectivity matrix was severely biased positively for about half of the subjects potentially due to global BOLD fluctuation [31] (S1 Fig). To address this, we controlled the effect of the mean signal of the constituent network signals for each subject by computing partial correlation [32–34] (S2 Fig). The distribution of the correlation between ROIs became more symmetric with the mean of zero (S3 Fig) and the expected anti-correlation between ROIs in the task-positive and the task-negative networks became more apparent than without controlling for mean signal (S1 and S2 Figs).

We first identified modular structures of connectivity for the two memory tasks. For this, we employed a module-detection algorithm to maximize modularity, which is a metric of functional segregation into distinct modules [35–37]. To consider variability of modular structures across subjects, we developed used a two-step probabilistic approach to the identification of modules. We first calculated “module-allegiance” which computes how frequently two ROIs are assigned to the same module. For each subject, task, and task-positive and task-negative network (i.e., the collection of all task-positive and task-negative ROIs), we ran Louvain module detection algorithm implemented in MATLAB (Mathworks Inc., MA, USA) [36] on the initially computed correlation matrix 1000 times with initial random assignment of 53 ROIs (task-positive) or 65 ROIs (task-negative) to 10 modules. This number of “starting” modules was arbitrarily selected to provide a sufficiently high number to potentially capture all relevant modules for the entire brain [38, 39], and the algorithm identified the actual number of detected modules in the given data. Then, we computed module allegiance between each pair of ROIs as the probability that two ROIs were assigned to the same module across the group of subjects, i.e., the fraction out of the overall 30,000 runs of the algorithm (1,000 per each of 30 subjects). This definition of module allegiance allows detection of maximum modularity, given that the modules identified by each run of the algorithm are not always identical. By definition of modularity, the assignment of modules tends to be more random for a network lower modularity (i.e., the detection algorithm is less reliable). Next, we re-ran the module detection algorithm on the module-allegiance matrix to identify the final modular structure of connectivity for the group of subjects.

Once we identified modular structures of connectivity, we calculated interactions among network components and correlated them with subsequent memory performance. Interaction was computed as mean correlation of all pairs of ROIs within and between networks and modules of the networks. We computed memory performance as the mean proportion of test trials with correct source memory responses and high-confidence correct recognition responses. We focused on this category because it is the most likely to reflect recollective memory without substantial contamination by guessing (see Results) and is specific to the source-memory information that differentiated the object-scene and object-location memory tasks. We first tested network-level interactions and then module-level interactions, using the modules that were detected as described above. P-values were Bonferroni corrected for multiple comparisons with the corrected significance level set to P_corr_ < 0.05. In the network-level analysis, there were three tests (POS-POS, NEG-NEG, POS-NEG) and in the module-level analysis, there were 10 tests for the scene-object task (4 modules: 6 between-module tests and 4 within-module tests) and 21 tests for the location-scene task (6 modules: 15 between-module tests and 6 within-module tests). These numbers of tests were used to determine the Bonferroni correction factors. Multiple linear regression was used to estimate the total variability in memory performance that could be accounted for by all network and module interactions that were individually significant predictors of memory accuracy. Brain images with activation maps were generated using MRIcroGL (http://www.mccauslandcenter.sc.edu).

We also compared the probabilistic connectivity method that we implemented to the more conventional averaging method [38] in terms of the ability for the detected networks and modules to significantly predict memory performance. That is, we investigated what extent was probabilistic connectivity analysis necessary for identifying connectivity patterns that robustly correlated with memory performance. In the average connectivity method, the constructed connectivity matrices (see above) were averaged across subjects and then modules were identified from the averaged connectivity matrix using the module detection algorithm.

## Results

### Memory performance

Subjects studied item-context associations using two formats of to-be-remembered stimuli (Fig 1). In the object-scene memory task, subjects studied arbitrary pairings of objects and scenes and memory was later tested for individual objects (recognition memory) as well as for the object-scene pairings (source memory). The object-location memory task was similar, but using objects arbitrarily paired with specific screen locations instead of with specific scenes. The tasks were matched in many characteristics in order to equate performance levels as closely as possible despite the differences in stimuli (see Materials and Methods).

**Fig 2:**
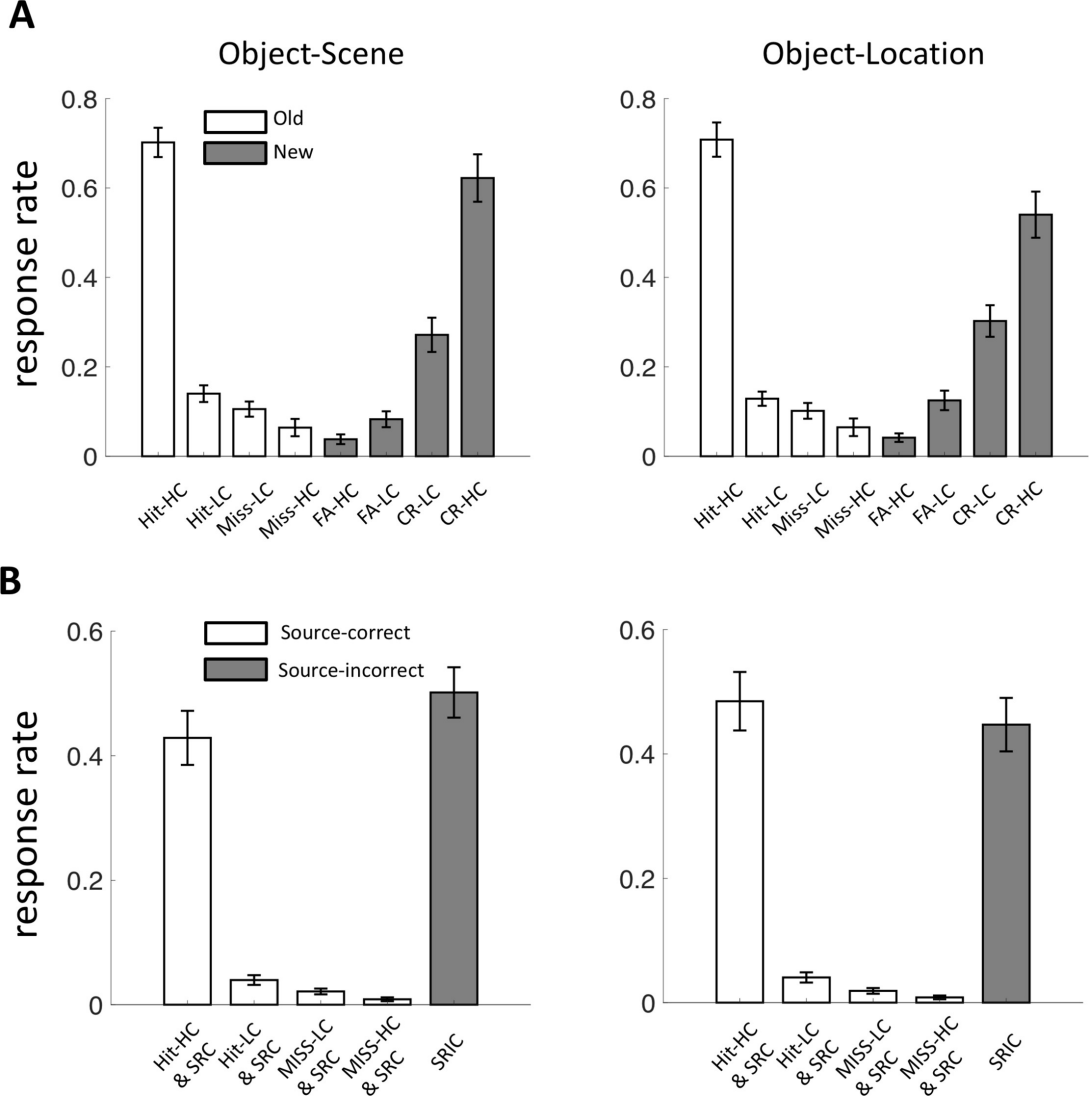
Memory performance. (A) Response rates for the recognition memory judgment are shown for the object-scene and object-location tasks, averaged separately for old versus new objects and for each confidence level (HC: high confidence; LC: low confidence) and each category of response accuracy (hit, miss, FA: false alarm, and CR: correct rejection). (B) Response rates for source memory judgments are shown averaged based on accuracy/confidence of the corresponding recognition response (SRC: source recollection correct, SRIC: source recollection incorrect). Trials with correct source recollection tended to include objects that were recognized with high confidence. Error bars indicate the standard error mean.

Recognition memory performance was highly accurate in the object-scene and object-location tests, as indicated by subjects’ ability to discriminate old from new objects at test. As indicated in Fig 2A, high-confidence “old” responses were primarily made to old items whereas high-confidence “new” responses were primarily made to new items, with intermediate levels of discrimination for lower confidence levels. Memory performance was defined as a rate of later-remembered trials after excluding the very few trials with missed responses (<1 on average, see Methods).

In an analysis of response rates during memory testing, the statistical interaction of memory status (old versus new stimuli) and response type (old versus new, each with four levels of confidence) was significant for both the object-scene and object-location tasks (F(3,87) = 5.76, P = 0.0012 and F(3,87) = 14.6, P < 0.001, respectively; two-way repeated-measures ANOVA), thereby suggesting successful memory performance in both tasks (Fig 2A). Source memory performance was also highly accurate in both tasks (Fig 2B). When considering trials with high-confidence recognition responses in order to reduce the possibility of correct recognition guessing, source memory responses were highly accurate (object-scene: 0.43 ± 0.044, object-location: 0.48 ± 0.047, mean ± SE), which were both significantly greater than the chance performance level of 1/6 (t(29) = 9.59, P < 0.0001 and t(29) = 11.39, P < 0.0001, respectively).

Memory performance in the object-scene and object-location tasks did not vary substantially. The statistical interaction of task type and response type was not significant (F(7,203) = 2.03, P = 0.053). Targeted analysis of pairwise differences in accuracy for each confidence level did not find any differences that survived correction for multiple comparisons. There were slightly higher rates of correctly endorsing new items as new with high-confidence (CR-HC) in the object-scene compared to object-location task (t(29) = 2.15, P = 0.040), but this did not survive correction for multiple comparisons. All other pairwise P values were > 0.081. Likewise, accuracy of source memory judgments did not differ significantly for the object-scene and object-location tasks regardless of recognition type (t(29) = 1.34, P = 0.19 for the trials with high-confidence recognition responses, t(29) = 1.47, P = 0.15 for all trials). Thus, comparisons of fMRI connectivity for the object-scene and object-location tasks were not confounded by overall differences in memory performance.

### Modular connectivity structure during study

**Fig 3:**
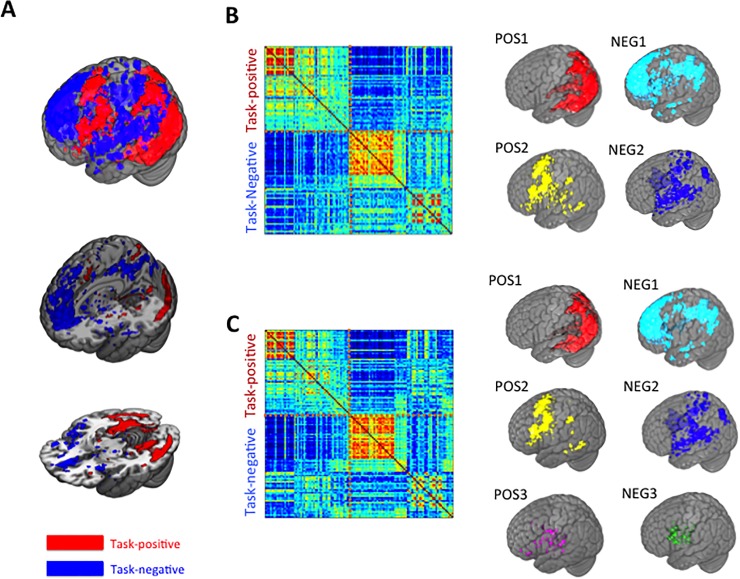
Task-related networks and modular structure. (A) Locations identified as task-positive (red) and task-negative (blue) (see Tables 1 and 2). These regions were identified via lenient univariate analysis coupled with anatomical demarcation for use as ROIs in subsequent connectivity analyses (see text). (B) Connectivity matrix for the object-scene task, sorted by identified modules, (Left) and the identified modules (Right) (C) Connectivity matrix for the object-location task, sorted according to the identified modules for the object-scene task (Left) and the identified modules (Right).

fMRI data acquired during the study phases of the memory tasks were analyzed in order to identify the modular structure of interregional fMRI connectivity patterns related to memory formation (see Materials and Methods). We defined regions of interest for fMRI connectivity analyses via group-level univariate analysis of stimulus-evoked activity with a liberal statistical threshold, which was divided and anatomically labeled by a structural atlas. This yielded a task-positive set of ROIs (53 ROIs, those that showed positive-going evoked activity deflections in response to all stimuli during study) as well as a task-negative set of ROIs (65 ROIs, regions with negative-going evoked activity deflections), as listed in Tables 1 and 2. As indicated in Fig 3A (see also S4 and S5 Figs), task-positive ROIs included visually sensitive dorsal and ventral areas including bilateral inferior temporal cortex, ventral occipito-temporal cortex, fusiform and lateral occipital cortex [40], parahippocampal cortex, and parietal cortex. In contrast, task-negative ROIs included orbitofrontal and medial prefrontal cortex, retrosplenial cortex, superior temporal cortex, anterior and posterior cingulate cortex, and parietal cortex, which largely overlapped with regions considered as part of the default mode network [41]. The spatial distribution of the task-positive and the task-negative ROIs is comparable to that reported in related studies [27, 31].

For each of ROIs, the spatially averaged fMRI time series was calculated in each subject for the study phase of each task and used for connectivity analysis. Comparison of module structure of the full ROI-to-ROI connectivity matrices using probabilistic module allegiance analysis (see Materials and Methods) indicated small yet significant differences between the two memory tasks (Fig 3B; Tables 1 and 2). For both tasks, the primary task-positive module corresponded to the dorsal/ventral visually responsive network (POS1 in Fig 3B and 3C). For the object-scene task, the second module mostly included fronto-parietal regions and fronto-limbic regions (fronto-parietal-limbic, POS2 in Fig 3B). For the object-location task, this module was fractionated into two modules, consisting of the separate fronto-parietal and fronto-limbic components (Fig 3C).

For both tasks, the primary task-negative module included orbital and medial prefrontal cortex, and anterior and posterior cingulate cortex areas of the default-mode network [41], as well as “extended” default-mode thalamic, striatal, and temporal-parietal cortical regions (default-extended-limbic, NEG1 in Fig 3B and 3C). For both tasks, the secondary task-negative module included inferior frontal regions, cuneus, subcortical areas including dorsal thalamus and cerebellum, and superior temporal and parietal cortex, which has been described as a “transitional” module, connecting cognition with emotion/interoception [42](NEG2 in Fig 3B and 3C). For the object-location task, this module was fractionated such that several subcortical regions formed a third module, including the limbic/paralimbic portions of the default-mode network (default-limbic, NEG3 in Fig 3C).

### Network interaction associated with memory formation

To investigate how the network-level (task-positive and task-negative networks: POS and NEG) and module-level interaction (4 modules for the object-scene task and 6 modules for the object-location task; see Tables 1 and 2) shown in Fig 3 correlated with memory performance, we first focused on source memory, as this type of memory varied in terms of demands between the two memory task formats (i.e., object-to-scene binding versus object-to-location binding). As indicated above, source memory performance was quantified as the proportion of trials with correct source-memory responses that also had high-confidence correct recognition memory responses (Fig 2B). Trials with low-confidence recognition responses were excluded because these were more likely to include recognition responses that were correct by guessing. Indeed, source memory accuracy was higher for high-confidence than low-confidence recognition responses (Fig 2B; object-scene: 0.43 ± 0.044 vs. 0.040 ± 0.0078, mean ± SE, t(29) = 8.59, P < 10^−8^, object-location: 0.48 ± 0.047 vs. 0.040 ± 0.0082, mean ± SE, t(29) = 8.95, P < 10^−9^). Additionally, source memory accuracy for trials with low-confidence recognition responses was significantly lower than the chance level of 1/6 (P< 10^−15^ for the two tasks), confirming that low-confidence recognition responses did not occur with reliable source memory. Finally, source memory accuracy with high-confidence recognition is highly correlated across subjects with recognition accuracy with high-confidence regardless of source-memory (correlation coefficient between object-scene: r = 0.81, object-location: r = 0.82. Therefore, we ignored confidence for recognition accuracy in the network interaction analysis, otherwise the results for recognition accuracy will be highly redundant with those for source memory accuracy.

Network-level interactions during memory formation predicting later source memory accuracy were evident within the task-positive network (R^2^ = 0.22, P_corr_ = 0.028), within the task-negative network (R^2^ = 0.34, P_corr_ = 0.0023), and between the task-positive and task-negative networks (R^2^ = 0.22, P_corr_ = 0.026), only for the object-scene task. All significant predictive relationships are summarized in Table 3. Network-level interactions did not significantly predict source memory performance for the object-location task. Thus, for the object-scene task, within-network interactions were positively correlated with memory scores whereas between-network interactions were negatively correlated with memory scores, suggesting that heightened interaction within task-positive and task-negative networks as well as anti-correlation of task-positive and task-negative networks were markers of successful memory formation.

**Table 3:** Summary of network interaction associated with memory formation.

	Network-level	Module-level
**Object-Scene task**	Source Memory	Source Memory
	1. POS-POS ρ = 0.47, R^2^ = 0.22, F(1,28) = 7.74, P_corr_ = 0.028	1. NEG1-NEG2 ρ = 0.71, R^2^ = 0.50, F(1,28) = 27.88, P_corr_ <0.001
	2. NEG-NEG ρ = 0.58, R^2^ = 0.34, F(1,28) = 4.29, P_corr_ = 0.0023	2. POS1-NEG2 ρ = -0.68, R^2^ = 0.46, F(1,28) = 23.96, P_corr_< 0.001
	3. POS-NEG ρ = -0.47, R^2^ = 0.22, F(1,28) = 7.96, P_corr_ = 0.026	3. POS1-POS1 ρ = 0.47, R^2^ = 0.23, F(1,28) = 8.14, P_uncorr_ = 0.0081
		4. POS2-NEG2 ρ = -0.38, R^2^ = 0.15, F(1,28) = 4.82, P_uncorr_ = 0.037
		5. Full-model ρ = 0.75, R^2^ = 0.57, F(4,25) = 8.28, P< 0.001
		6. Model (1+2) ρ = 0.74, R^2^ = 0.54, F(2,27) = 15.92, P< 10–4
		Non-significant interactions: 0.05 < P_uncorr_ < 0.80
	Recognition Memory	Recognition Memory
	None	1. POS1-NEG2 ρ = -0.57, R^2^ = 0.32, F(1,28) = 13.27, P_corr_ = 0.011
		2. NEG1-NEG2 ρ = 0.42, R^2^ = 0.18, F(1,28) = 6.17, P_uncorr_ = 0.019
		3. Full-model ρ = 0.57, R^2^ = 0.32, F(2,27) = 6.42, P = 0.0052
		Non-significant interactions: 0.10 < P_uncorr_ < 0.93
	Source memory controlling recognition memory	Source memory controlling recognition memory
	1. NEG-NEG ρ = 0.51, R^2^ = 0.26, P = 0.0049	1. NEG1-NEG2 ρ = 0.65, R^2^ = 0.43, P <0.001
	2. POS-NEG ρ = -0.37, R^2^ = 0.14, P = 0.047	2. POS1-NEG2 ρ = -0.46, R^2^ = 0.21, P = 0.011
		3. POS1-POS1 ρ = 0.40, R^2^ = 0.16, P = 0.033
		4. POS2-NEG2 ρ = -0.52, R^2^ = 0.27, P = 0.0037
	Source Memory	Source Memory
	None	1. POS2-POS2 ρ = -0.58, R^2^ = 0.34, F(1,28) = 14.11, P_corr_ = 0.017
		2. POS2-NEG3 ρ = -0.49, R^2^ = 0.24, F(1,28) = 8.63,P_uncorr_ = 0.0065
		3. Full-model ρ = 0.63, R^2^ = 0.39, F(2,27) = 8.74, P = 0.0012
		Non-significant interactions: 0.05 < P_uncorr_ < 1.00
**Object-Location task**	Recognition Memory	Recognition Memory
	None	1. POS2-POS2 ρ = -0.63, R^2^ = 0.40, F(1,28) = 18.72, P_corr_ = 0.0037
		2. POS1-POS2 ρ = 0.41, R^2^ = 0.17, F(1,28) = 5.78, P_uncorr_ = 0.023
		3. Full-model ρ = 0.66, R^2^ = 0.43, F(2,28) = 10.17, P< 0.001
		Non-significant interactions: 0.06 < P_uncorr_ < 0.93
** **	Source memory controlling recognition memory	Source memory controlling recognition memory
	None	1. POS2-NEG3 ρ = -0.37, R^2^ = 0.14, P = 0.050

**Fig 4:**
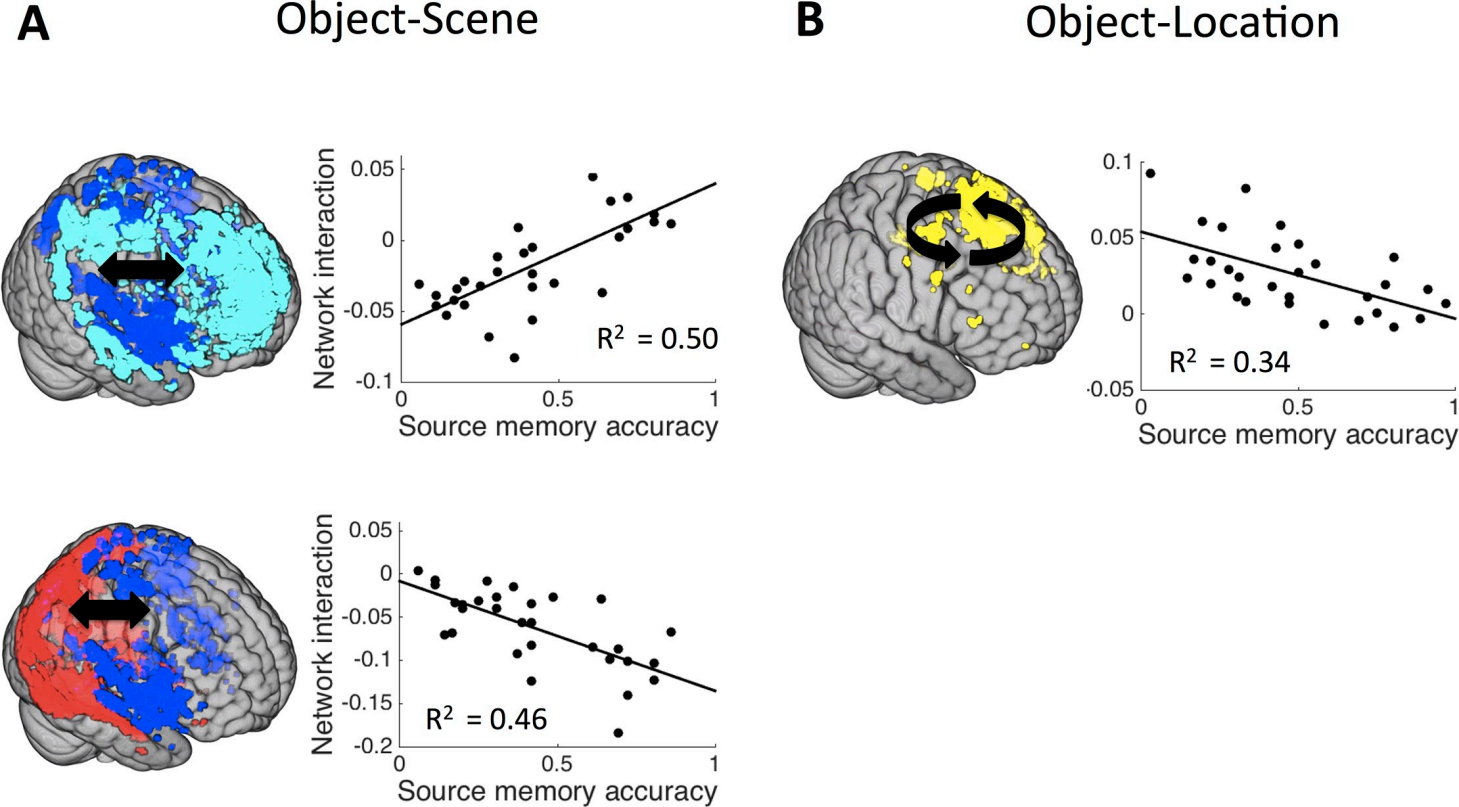
Correlation of source memory accuracy with module-level interaction. (A) For object-scene association task, interaction between two task-negative modules shows positive correlation (R^2^ = 0.50, P_corr_ < 0.001) and interaction between task-positive module and task-negative module shows negative correlation (R^2^ = 0.46, P_corr_ < 0.001). (B) For object-location association task, interaction within a task-positive module shows negative correlation (R^2^ = 0.34, P_corr_ = 0.017).

Module-level interactions predictive of subsequent source memory were assessed next. For the object-scene task the interaction of two modules of the task-negative network (NEG1-NEG2) was positively correlated with the memory scores, R^2^ = 0.50, P_corr_ < 0.001 (upper panel in Fig 4A). Additionally, interaction of the dorsal/ventral visual module of the task-positive network and the secondary module of the task-negative network (POS1-NEG2) was negatively correlated with the memory scores, R^2^ = 0.46, P_corr_ < 0.001 (lower panel in Fig 4A). Thus, for the object-scene task, increased interaction among task-negative modules and decreased interaction of task-negative and task-positive modules predicted successful memory formation. For the object-location task, interaction within the fronto-parietal module of the task-positive network (POS2-POS2) was negatively correlated with the memory scores, R^2^ = 0.34, P_corr_ = 0.017 (Fig 4B). There were three other interactions, two for the object-scene task (POS1-POS1: R^2^ = 0.23, P_uncorr_ = 0.0081; POS2-NEG2: R^2^ = 0.15, P_uncorr_ = 0.037) and one for the object-location task (POS2-NEG3: R^2^ = 0.24, P_uncorr_ = 0.0065), which were significant uncorrected (P_uncorr_ < 0.05) but did not survive Bonferroni correction (see Table 3 for details).

To determine the overall variance in source memory that could be accounted for by connectivity, we constructed a multiple linear regression model for each of the tasks including all significant interactions as predictors. The object-scene task model included four significant predictors and yielded R^2^ = 0.57, P < 0.001. The object-location task model included two significant predictors and yielded R^2^ = 0.39, P = 0.0012. A reduced model of the object-scene task including only the two most significant predictors (interactions, NEG1-NEG2 and POS1-NEG2) accounted for almost as much variance as the full model including four predictors (R^2^ = 0.54, P < 10^−4^). A large portion of the across-subject variance in source memory performance could thus be accounted for via network and module interconnectivity, with more variance in object-scene than object-location performance accounted for by interconnectivity.

The goal of this study was not to dissociate source memory from recognition memory, and in fact these were highly correlated in the current data (source memory accuracy vs. recognition memory accuracy: r = 0.74 averaged for both tasks). Nevertheless, we separately tested for relationships between connectivity and recognition. In general, connectivity was less predictive of recognition memory than it was for source memory, as would be expected due to the fact that the object memory demands were mostly similar between the two task formats. Recognition memory was quantified as the proportion of later-correct trials during study irrespective of response confidence. For both tasks, the network-level interactions were not significantly related to recognition memory (P_uncorr_ > 0.05). Some module-level interactions identified in the source-memory analysis were significant in the recognition-memory analysis, but to a lesser extent. The interaction of dorsal/ventral visual module with the secondary task-negative module (POS1-NEG2) for the object-scene task was negatively correlated with recognition memory (R^2^ = 0.32, P_corr_ = 0.011). The interaction of the two negative modules (NEG1-NEG2) was also positively correlated with recognition memory, but this relationship did not survive correction for multiple comparisons (R^2^ = 0.18, P_corr_ > 0.05, P_uncorr_ = 0.019). For the object-location task, the within-module interaction of the fronto-parietal module of the task-positive network (POS2-POS2) was negatively correlated with recognition memory (R^2^ = 0.40, P_corr_ = 0.0037), as was the case for source memory. Interaction between the two task-positive modules, dorsal/ventral visual module and fronto-parietal-limbic module (POS1-POS2) was also positively significant but did not survive Bonferroni correction (R^2^ = 0.17, P_corr_ > 0.05, P_uncorr_ = 0.023).

To test for the variance in recognition memory that could be accounted for by connectivity during study, we constructed a multiple linear regression model combining the two significant interactions for each of tasks. In contrast to the result for source memory, the performance of the model was lower for the object-scene (R^2^ = 0.32, P = 0.0052) task than for the object-location task (R^2^ = 0.43, P < 0.001). Thus, the network-level and module-level interactions predictive of source recollection memory also accounted for recognition memory although the interactions accounted for less variance in recognition memory than in source memory, especially for the object-scene task.

We also tested whether connectivity was still predictive of source memory accuracy when controlling for recognition memory. This post-hoc analysis indicated that network-level interactions survived within the task-negative network (R^2^ = 0.26, P = 0.0049) and between the task-positive and task-negative network (R^2^ = 0.14, P = 0.047) only for the object-scene task. Module-level interactions were likewise still predictive of source memory scores in two modules of the task-negative network (NEG1-NEG2, R^2^ = 0.43, P < 0.001) and in the dorsal/ventral visual module of the task-positive network and the secondary module of the task-negative network (POS1-NEG2, R^2^ = 0.21, P = 0.011). All other interactions are summarized in Table 3. For the object-location task, network-level and module-level interactions were non-significant when controlling for recognition memory scores except for the module-level interaction, POS2-NEG3, that was of borderline significance in the main analysis (Table 3).

To summarize, there were no relationships between connectivity and recognition that were not also identified for source recollection, relationships were more robust for source recollection, and controlling for recognition generally weakened correlations related to source recollection. Collectively, this indicates that memory-related connectivity was not qualitatively distinct for recollection versus recognition, but was quantitatively greater for recollection.

### Comparison with the averaged-connectivity method

To evaluate whether probabilistic connectivity analysis aided identification of modules related to memory formation relative to the standard, averaged-connectivity method, we performed the same analyses described above but using averaged-connectivity (see Materials and Methods). Connectivity structure assessed with the averaged connectivity was similar to that identified using the probabilistic method but with less clear distinction of modules (S6 Fig). For the object-scene task, a limbic module was identified that was distinct from the fronto-parietal-limbic module (POS2) and the default-extended-limbic module (NEG1), which were treated as one large module using the probabilistic method. Thus, as was the case for the object-location task, there were three modules for each of the task-positive and negative networks when identified using the averaged-connectivity method. For the location task, the number of identified modules was same as identified using the probabilistic method. However, some ROIs were assigned to different modules across the two methods (10 of 53 ROIs and 3 of 65 ROIs for the task-positive and task-negative networks were assigned to different modules based on the two methods).

More importantly, similar interactions were predictive of source memory and recognition memory in the object-scene but not in the object-location task. For the object-scene task, two similar module-level interactions were significantly predictive of source memory, but with slightly reduced correlation values (R^2^ = 0.48, R^2^ = 0.42) relative to the values obtained using the probabilistic-connectivity method (R^2^ = 0.50, 0.46, see Fig 4A). The interaction between the dorsal/ventral visual module of task-positive network (POS1) and the secondary task-negative module (NEG2) was negatively predictive of recognition memory accuracy (R^2^ = 0.34), similar to results from the probabilistic method (see Table 3). For the object-location task, however, no modular interactions significantly correlated with source memory accuracy or recognition memory accuracy using the averaged-connectivity method. The within-module fronto-parietal (POS2) interaction predicting source memory (Fig 4B), was not significant using averaged connectivity, likely because ROIs driving this relationship were assigned to different modules relative to the probabilistic-connectivity method (S6 Fig).

## Discussion

We investigated functional brain networks during two different item-context memory tasks and their interactions associated with successful memory formation. We used a relatively novel method of probabilistically defining connectivity between regions such that interindividual variability can be conserved in a network structure for a group of subjects. This probabilistic method of detecting modules using “module-allegiance” is potentially superior to the conventional method using averaged connectivity for group analysis because it conserves individual differences in network topology, whereas the averaging method could eliminate individual variability [20]. Here, the probabilistic method was more successful at discriminating differences in network structure between the memory tasks compared to the conventional averaging method. Furthermore, the modules detected by the probabilistic method were more informative than those detected by the averaging method in terms of identifying modules with interactions that significantly accounted for variability of memory performance.

Networks varied in functional modules for the object-scene and object-location tasks. However, the overall network structures were quite similar. This is not surprising as previous findings suggest high similarities of network architecture across highly distinct tasks and resting-state fMRI [43, 44]. Such previous findings have led to the suggestion that task-related functional networks are primarily shaped by intrinsic/structural connectivity with only a limited set of connectivity patterns changing due to cognitive demands. Nonetheless, the small yet significant differences in network structure between tasks were important for understanding brain-behavior relationships. That is, network and module-level interactions differentially predicted performance in the two tasks, and therefore accurate quantification of the specific connectivity patterns unique to each task was essential for identifying the relationship between these interactions and memory formation. This was true despite the fact that both were episodic memory tasks sharing a large degree of demand characteristics.

Despite relatively high overall similarity in network structure, the interaction of functional networks and modules differently predicted memory accuracy in the object-scene versus object-location tasks. Our results show that memory performance could be mediated by cooperative interaction (i.e., functional coupling) within or between modules in the same network, either task-positive (Fig 4B) or task-negative (upper in Fig 4A), and competitive interaction (i.e., functional de-coupling) between modules in the different networks (lower in Fig 4A). These results are consistent with previous findings reporting connectivity-behavior relationships in cognitive tasks such as working memory [28, 45–47], visual discrimination [48], reading competence [49], variability of reaction time [26], and task automatization [50]. Specifically, in one study by Hampson et al [45], task demands and individual working memory performance were significantly positively correlated with the connectivity among posterior cingulate cortex, medial frontal cortex, and ventral anterior cingulate cortex—all regions of the task-negative default-mode network [45]. Other findings suggest that interaction of the task-positive working-memory network and task-negative default-mode network is negatively correlated with working memory performance [28, 46]. Resting-state connectivity is similarly related to visual discrimination performance [48] and reading competence [49]. In those studies, positive connectivity among task-positive regions associated with visual perception and reading and negative connectivity of these regions with task-negative default-mode regions predicted visual discrimination and reading performance, respectively. Likewise, stronger negative correlation between task-positive and task-negative regions has been associated with greater stability of cognitive function [26]. Recently, similar interactions within and between task-positive and task-negative networks have been related to short-term task automatization and proposed as a general property by which large-scale networks reconfigure to meet task demands [50]. These properties were especially important for object-scene memory formation relative to object-location memory formation, again suggesting that these different memory tasks require substantially different cognitive and neural resources despite their superficial similarities.

In the current data, relationships between network interactions and memory formation were generally less robust for the object-location task than they were for the object-scene task. For example, in contrast to the object-scene findings, there was no network-level interaction accounting for memory accuracy in the object-location task. At the module level, only one within-module interaction of the task-positive network was associated with memory performance for the object-location task, which was less significant compared to relationship identified for the object-scene task (Fig 4B). Moreover, the overall ability for a model with multiple regressors to account for total variation in memory performance across subjects was much lower in the object-location task than in the object-scene task. Thus, even though these tasks shared much in common, including overall similar functional network architectures, our findings suggest that they are nonetheless mediated by different types of network interactions.

It is highly likely that the cognitive operations required for object-location and object-scene memory formation are fundamentally different, and we speculate that the nature of these differences might have been related to the observed distinctions in network interactions. For the object-location memory task, only a single object was presented on the screen and performance may have been supported by detailed attention to each visual stimulus with respect to the perceptual details of its specific screen location (see [51] for relevant discussion). This account is consistent with our finding that performance was predicted by increased coupling of task-positive regions, which may have reflected heightened focus on visual information. In contrast, the object-scene task required arbitrary relational binding of two simultaneously presented stimuli. Subjects might therefore have internally generated meaningful links between the stimuli (i.e., semantic associations) to a greater extent than in the object-location task. This distinction could be considered to reflect relatively more associative versus “unitized” representations[52] in the object-scene versus object-location tasks, respectively. This is consistent with our finding that object-scene performance was predicted by relative decoupling of task-positive and task-negative regions, which could reflect engagement of cognitive strategies that occur with relative disengagement from the visual stimulus processing. Of course, it is also possible that nonspecific factors (i.e., arousal, vigilance, and attention) are responsible for relationships between fMRI connectivity and specific behavioral measures such as those used to measure memory. However, it is highly unlikely that such nonspecific factors could have produced differences in connectivity-performance relationships between the object-scene and object-location tasks, as similar levels of performance and task demands guard against such possibility. Thus, we consider it more likely that differences in connectivity patterns related to memory performance between tasks were due to specific cognitive demands that varied between tasks.

Different types of network interactions could also be relevant for memory formation versus memory retrieval. For instance, a recent study found that accurate memory retrieval was associated with higher interaction between a task-positive frontoparietal control network and a task-negative default mode network [53]. The two networks do not strongly interact in most cognitive tasks, and they are typically anti-correlated (as was the case in our data). Indeed, we found that, in general, greater anti-correlation of task-positive and task-negative network components correlated with better memory formation, at least for the object-scene task. Clearly, cognitive demands differ for memory formation and memory retrieval, with retrieval perhaps requiring greater recruitment of task-positive networks for strategic processing of the contents of task-negative core memory networks. In contrast, memory formation might be benefitted by heightened internally focused processing, as could support episodic and semantic elaboration and other cognitive operations that could increase the richness of memory formation. This distinction between demands of memory formation versus memory retrieval could thus relate to the different patterns of network segregation as related to memory success that were identified in our study of memory formation versus other findings of memory retrieval [40]. Future research could directly compare network interactions between encoding and retrieval within the same task to substantiate encoding/retrieval similarities or differences in network interactions suggested by the current findings [54]

Although the current findings highlight large-scale brain network interactions for memory formation and indicate that networks reconfigure to support different memory-formation demands, it remains to be seen whether there are any general operating principles of such functional reorganization for memory. By using methods such as those reported here to better capture individual differences in functional brain networks, general principles of network re-organization for episodic memory could be best identified and compared to principles operative for other cognitive domains.

## Supporting information

S1 Fig Connectivity matrix of 30 subjects using simple correlation without mean signal correction. For some subjects, task-positive and task-negative networks were observed, as apparent by their anti-correlation (i.e., upper-left and lower-right of each graph). However, global BOLD signal fluctuation potentially positively biased correlation between ROIs for other subjects such that task-positive and task-negative networks could not be discerned. We addressed this issue by controlling the mean signals over the entire brain (S2 Fig).
(TIFF)

S2 Fig Connectivity matrix of 30 subjects using partial correlation controlling the mean signal. The positively biased correlation was reduced by controlling the mean signal. The task-positive and the task-negative networks were more apparent in every subject when partial correlation controlling mean signal was used versus simple correlation (S1 Fig).
(TIFF)

S3 Fig Distribution of connectivity across all the nodes, conditions (object-scene and object-location tasks), and subjects. (A) Distribution of simple correlation values. (B) Distribution of the same values but with partial correlation used to control the mean network component signal.
(TIFF)

S4 Fig Activation map of task-positive and task-negative responses. Supplementary activation map to Fig 3 showing the spatial distribution of the task-positive and the task-negative networks. We used a lenient voxel-wise P < 0.05 for network construction and thus make no statistical significance of this activation map. Brain images with activation maps were generated by publicly available software, MRIcroGL (http://www.mccauslandcenter.sc.edu).
(TIFF)

S5 Fig An example showing how anatomical ROIs were separated into two functional ROIs. This example frontal region defined by anatomical atlas included both of task-positive (red) and task-negative (blue) voxels. The region in green was discarded because the voxels are neither of task-positive or task-negative i.e., t(29) > 0.05, with the regions that were significantly task-responsive segregated into two ROIs based on whether their responses were task-positive or task-negative. Brain images with activation maps were generated by publicly available software, MRIcroGL (http://www.mccauslandcenter.sc.edu).
(TIFF)

S6 Fig Comparison of two methods for identifying modular structure. The probabilistic method was superior to the conventional averaging method in terms of its ability to identify modules with interactions that were significantly correlated with memory task performance. Modules were also more visually apparent when identified with the probabilistic method compared to the averaged method (for comparison, all the connectivity matrices were ordered according to the identified modules using the probabilistic method).
(TIFF)

S1 Table Labels of original 85 ROIs. (DOCX)

S2 Table Description of final 118 ROIs. (DOCX)
