# Correction: Large-scale network interactions supporting item-context memory formation

**DOI:** 10.1371/journal.pone.0216195

**Published:** 2019-04-24

**Authors:** Sungshin Kim, Joel L. Voss

The following information is missing from the Funding statement: This research is supported by the Institute of Basic Sciences, Republic of Korea, grant number IBS-R015-D1.
