# Supplementary figures and images for: Large-scale network interactions supporting item-context memory formation
Source: PLoS One. 2019 Jan 10;14(1):e0210167. doi: 10.1371/journal.pone.0210167 (PMC6328164; doi:10.1371/journal.pone.0210167)

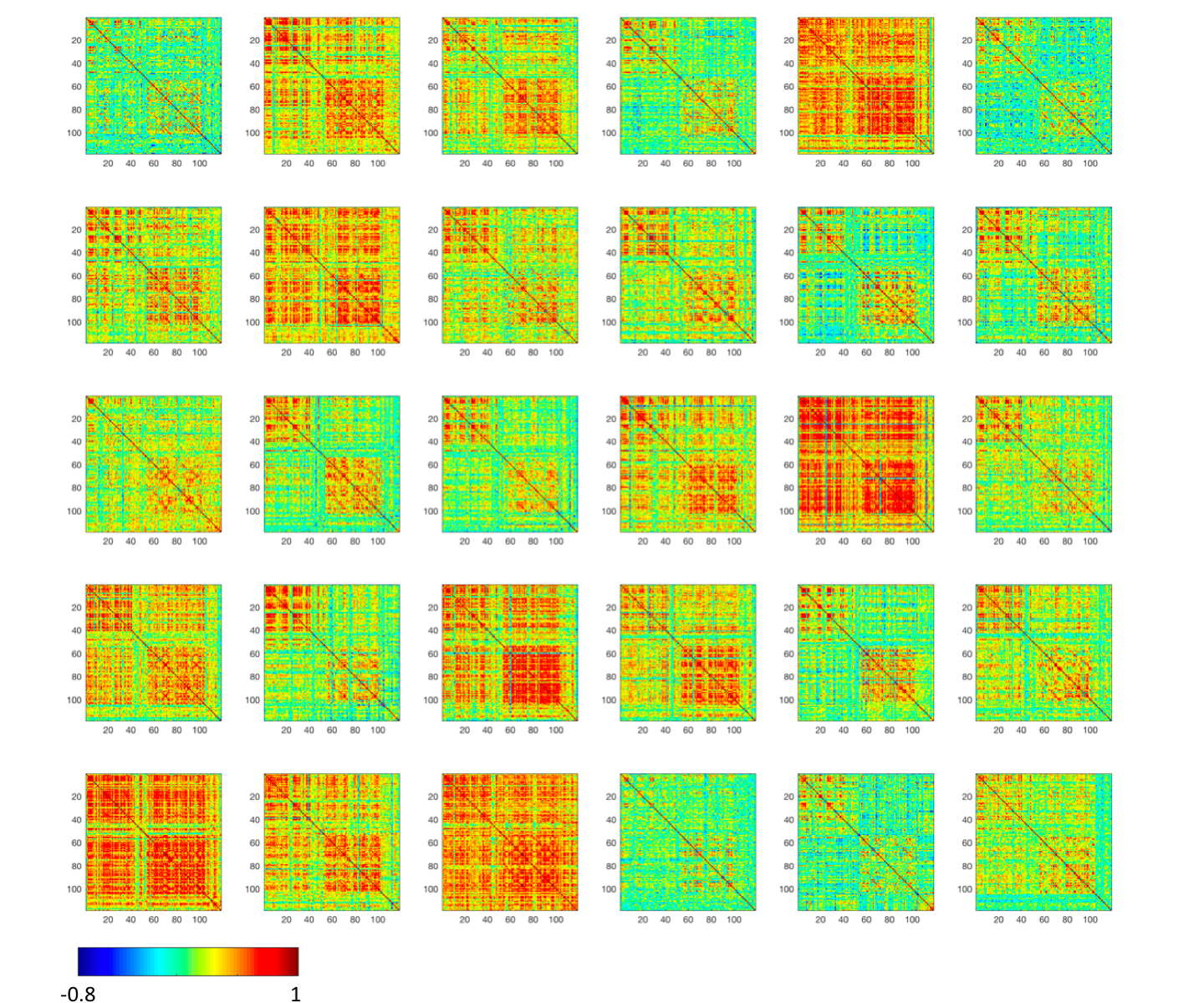

Supplement: S1 Fig — For some subjects, task-positive and task-negative networks were observed, as apparent by their anti-correlation (i.e., upper-left and lower-right of each graph). However, global BOLD signal fluctuation potentially positively biased correlation between ROIs for other subjects such that task-positive and task-negative networks could not be discerned. We addressed this issue by controlling the mean signals over the entire brain (S2 Fig). (TIFF) [file pone.0210167.s001.tiff]

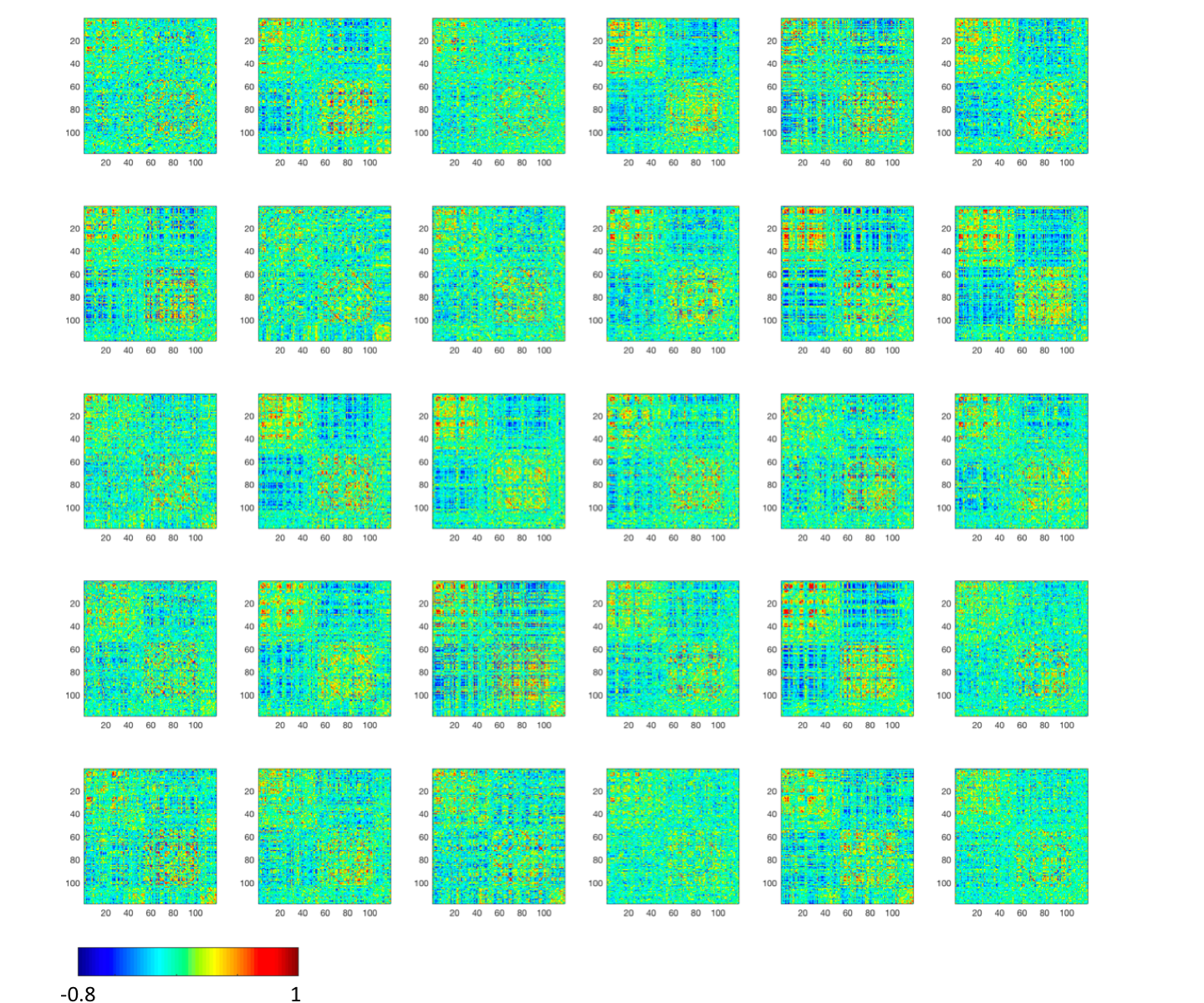

Supplement: S2 Fig — The positively biased correlation was reduced by controlling the mean signal. The task-positive and the task-negative networks were more apparent in every subject when partial correlation controlling mean signal was used versus simple correlation (S1 Fig). (TIFF) [file pone.0210167.s002.tiff]

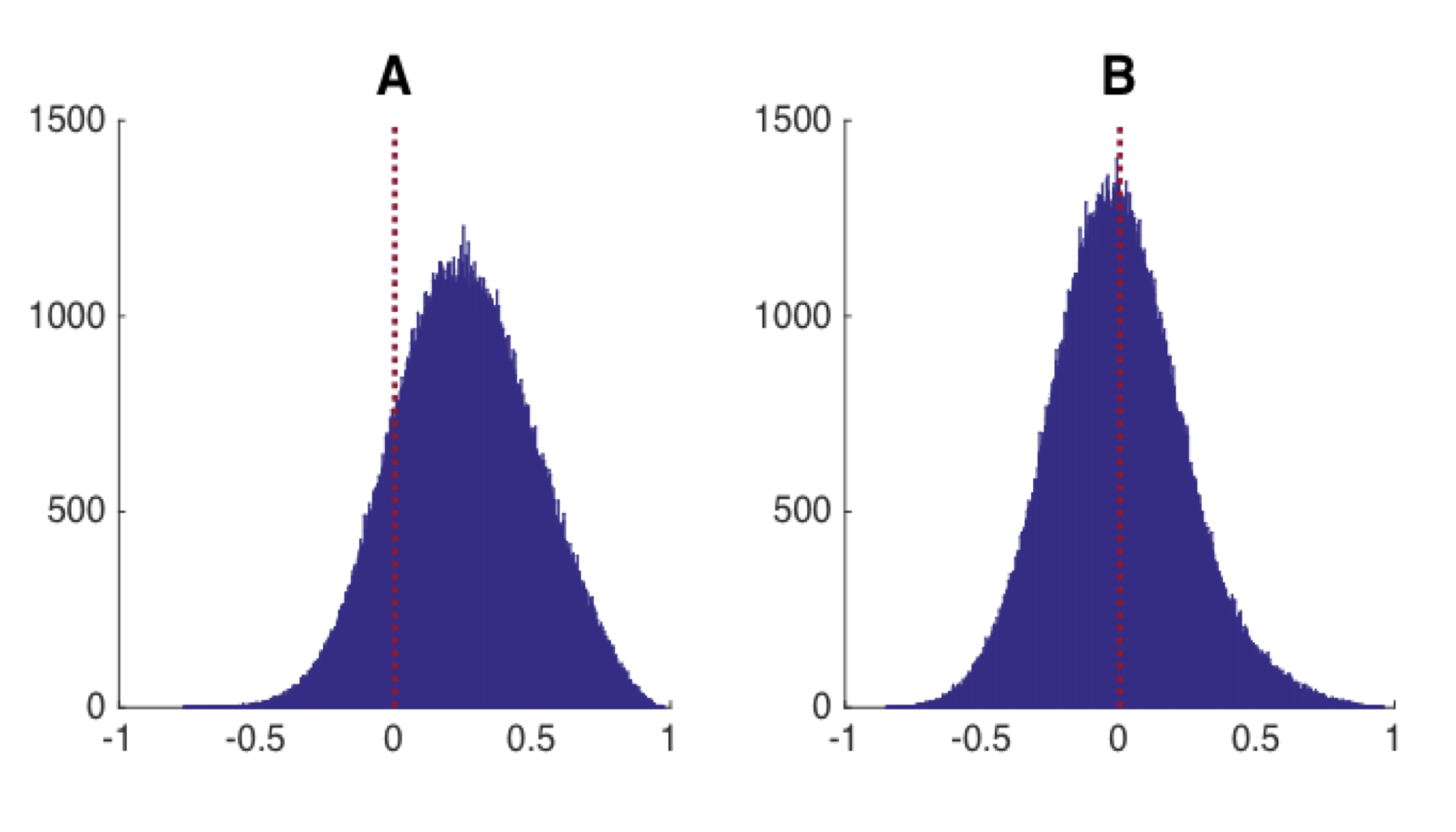

Supplement: S3 Fig — (A) Distribution of simple correlation values. (B) Distribution of the same values but with partial correlation used to control the mean network component signal. (TIFF) [file pone.0210167.s003.tiff]

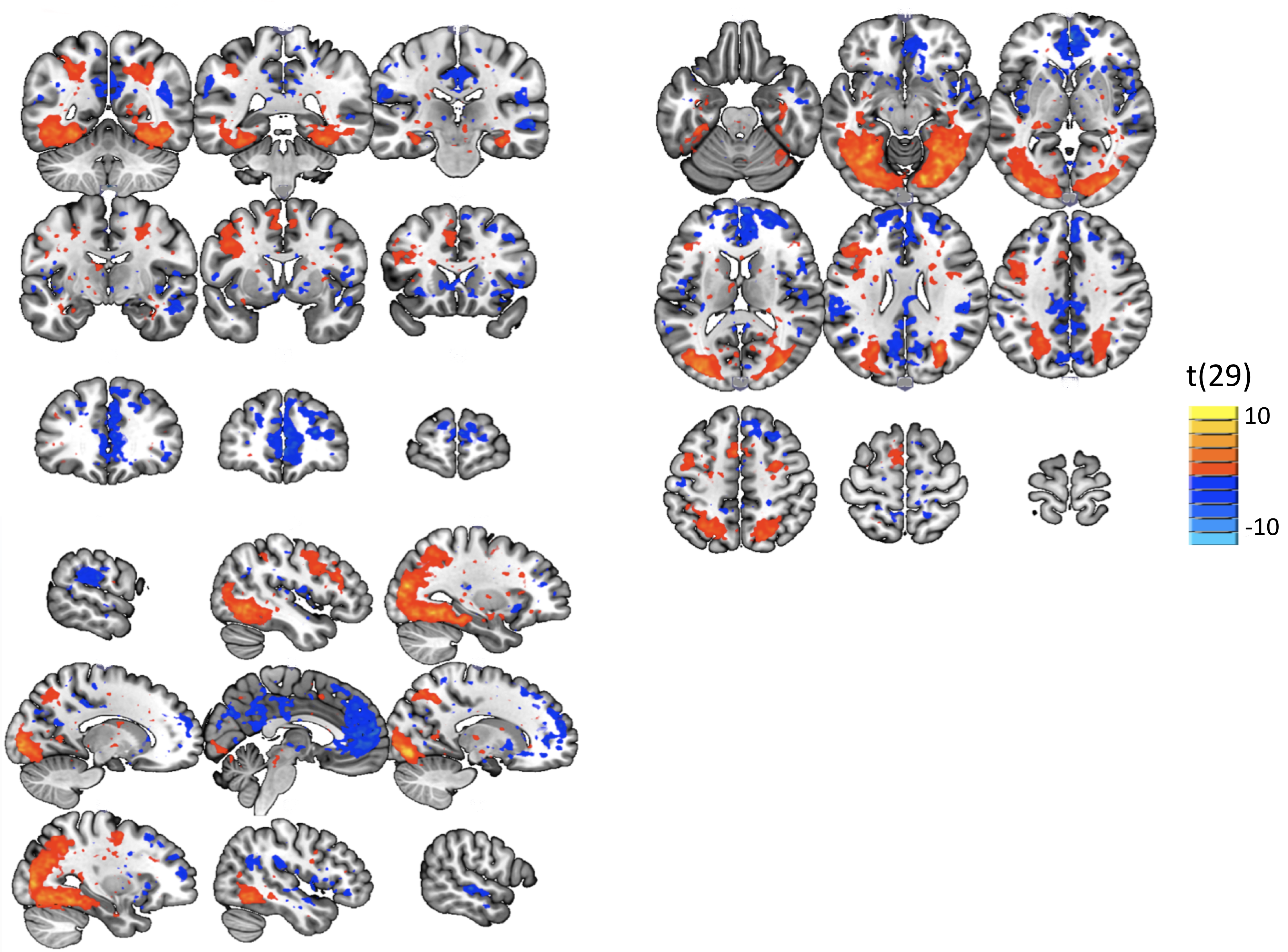

Supplement: S4 Fig — Supplementary activation map to Fig 3 showing the spatial distribution of the task-positive and the task-negative networks. We used a lenient voxel-wise P < 0.05 for network construction and thus make no statistical significance of this activation map. Brain images with activation maps were generated by publicly available software, MRIcroGL (http://www.mccauslandcenter.sc.edu). (TIFF) [file pone.0210167.s004.tiff]

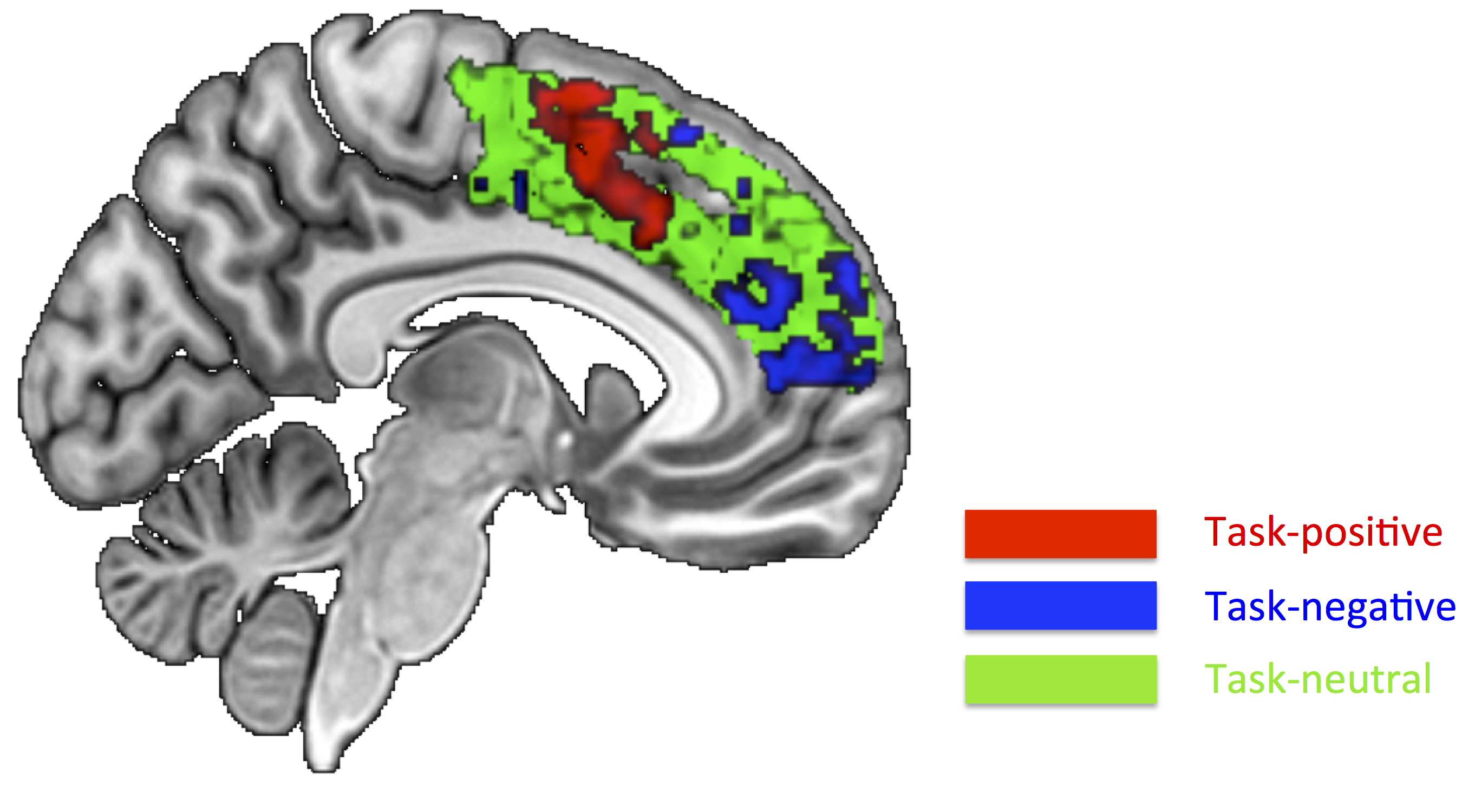

Supplement: S5 Fig — This example frontal region defined by anatomical atlas included both of task-positive (red) and task-negative (blue) voxels. The region in green was discarded because the voxels are neither of task-positive or task-negative i.e., t(29) > 0.05, with the regions that were significantly task-responsive segregated into two ROIs based on whether their responses were task-positive or task-negative. Brain images with activation maps were generated by publicly available software, MRIcroGL (http://www.mccauslandcenter.sc.edu). (TIFF) [file pone.0210167.s005.tiff]

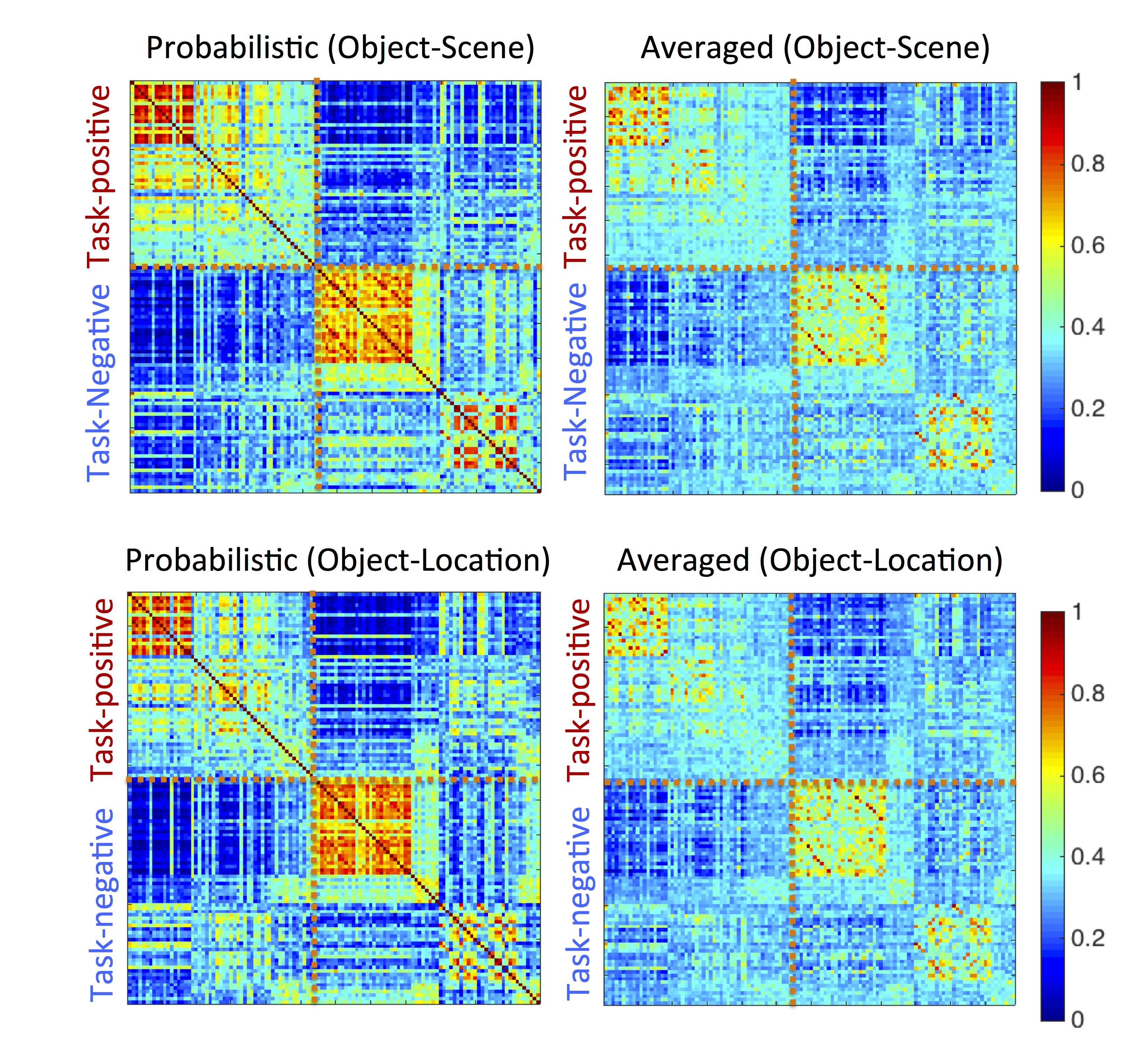

Supplement: S6 Fig — The probabilistic method was superior to the conventional averaging method in terms of its ability to identify modules with interactions that were significantly correlated with memory task performance. Modules were also more visually apparent when identified with the probabilistic method compared to the averaged method (for comparison, all the connectivity matrices were ordered according to the identified modules using the probabilistic method). (TIFF) [file pone.0210167.s006.tiff]
