# Supplementary material for: Large-scale network interactions supporting item-context memory formation
Source: PLoS One. 2019 Jan 10;14(1):e0210167. doi: 10.1371/journal.pone.0210167 (PMC6328164; doi:10.1371/journal.pone.0210167)
Supplement: S1 Table — (DOCX) [file pone.0210167.s007.docx]

**S1 Table. Labels of original 85 ROIs**

**A. Cortical ROIs**

| **Left hemisphere (34 regions)** | **Right hemisphere (36 regions)** |
| --- | --- |
| L-banks of superior temporal sulcus  L-caudal anterior cingulate  L-caudal middle frontal  L-cuneus  L-entorhinal  L-fusiform  L-inferior parietal  L-inferior temporal  L-isthmuscingulate  L-lateral occipital  L-lateral orbitofrontal  L-lingual  L-medial orbitofrontal  L-middle temporal  L-parahippocampal  L-paracentral  L-pars opercularis  L-pars orbitalis  L-pars triangularis  L-pericalcarine  L-postcentral  L-posterior cingulate  L-precentral  L-precuneus  L-rostral anterior cingulate  L-rostral middle frontal  L-superior frontal  L-superior parietal  L-superior temporal  L-supramarginal  L-frontal pole  L-temporal pole  L-transverse temporal  L-insula | R-banks of superior temporal sulcus  R-caudal anterior cingulate  R-caudal middle frontal  R-cuneus  R-entorhinal  R-fusiform  R-inferior parietal  R-inferior temporal  R-isthmuscingulate  R-lateral occipital  R-lateral orbitofrontal  R-lingual  R-medial orbitofrontal  R-middle temporal  R-parahippocampal  R-paracentral  R-pars opercularis  R-pars orbitalis  R-pars triangularis  R-postcentral  R-posterior cingulate  R-precentral  R-precuneus  R-rostral anterior cingulate  R-rostral middle frontal  R-superior frontal  R-superior parietal  R-superior temporal  R-supramarginal  R-frontal pole  R-temporal pole  R-transverse temporal  R-insula  R-accumbens  R-ventral DC  R-hippocampus |

**B. Subcortical ROIs**

| **Left hemisphere (9 regions)** | **Right hemisphere (6 regions)** |
| --- | --- |
| L-cerebellar cortex  L-dorsal thalamus  L-caudate  L-putamen  L-pallidum  L-amygdala  L-accumbens  L-ventral DC  L-hippocampus | R-cerebellar cortex  R-dorsal thalamus  R-caudate  R-putamen  R-pallidum  R-amygdala |

**Note: L – Left hemisphere, R – Right hemisphere**
