# Supplementary material for: Large-scale network interactions supporting item-context memory formation
Source: PLoS One. 2019 Jan 10;14(1):e0210167. doi: 10.1371/journal.pone.0210167 (PMC6328164; doi:10.1371/journal.pone.0210167)
Supplement: S2 Table — (DOCX) [file pone.0210167.s008.docx]

**S2 Table. Description of final 118 ROIs**

**A. Task-positive ROIs**

| **No.** | **Label** | **MNI coordinate**  **X Y Z** | | | **Number of voxels** |
| --- | --- | --- | --- | --- | --- |
| 1 | L-bankssts | 60 | 43 | -6 | 11 |
| 2 | L-caudal middle frontal | 45 | 1 | 46 | 583 |
| 3 | L-cuneus | 2 | 82 | 3 | 57 |
| 4 | L-fusiform | 35 | 50 | -26 | 1563 |
| 5 | L-inferior parietal | 37 | 85 | 13 | 1393 |
| 6 | L-inferior temporal | 52 | 55 | -24 | 892 |
| 7 | L-lateral occipital | 35 | 87 | -13 | 2717 |
| 8 | L-lateral orbitofrontal | 30 | -37 | -12 | 38 |
| 9 | L-lingual | 14 | 65 | -19 | 240 |
| 10 | L-middle temporal | 59 | 55 | -12 | 127 |
| 11 | L-parahippocampal | 26 | 29 | -24 | 26 |
| 12 | L-pars opercularis | 51 | -15 | 24 | 247 |
| 13 | L-pars orbitalis | 38 | -38 | -13 | 27 |
| 14 | L-pars triangularis | 49 | -28 | 10 | 119 |
| 15 | L-postcentral | 52 | 30 | 34 | 22 |
| 16 | L-precentral | 50 | 6 | 39 | 525 |
| 17 | L-precuneus | 4 | 69 | 24 | 78 |
| 18 | L-rostral middlefrontal | 48 | -23 | 30 | 364 |
| 19 | L-superior frontal | 7 | -4 | 57 | 1006 |
| 20 | L-superior parietal | 28 | 72 | 42 | 1668 |
| 21 | L-supramarginal | 50 | 49 | 39 | 167 |
| 22 | L-insula | 35 | -12 | 2 | 46 |
| 23 | R-caudal anterior cingulate | -4 | -23 | 29 | 10 |
| 24 | R-caudal middle frontal | -34 | 8 | 53 | 109 |
| 25 | R-entorhinal | -28 | 7 | -35 | 11 |
| 26 | R-fusiform | -34 | 45 | -26 | 1693 |
| 27 | R-inferior parietal | -36 | 81 | 16 | 448 |
| 28 | R-inferior temporal | -50 | 51 | -23 | 213 |
| 29 | R-lateral occipital | -32 | 85 | -15 | 909 |
| 30 | R-lateral orbitofrontal | -28 | -30 | -17 | 10 |
| 31 | R-lingual | -15 | 69 | -20 | 358 |
| 32 | R-parahippocampal | -26 | 30 | -22 | 96 |
| 33 | R-pars triangularis | -50 | -31 | 14 | 17 |
| 34 | R-precentral | -41 | 10 | 42 | 89 |
| 35 | R-precuneus | -10 | 67 | 26 | 112 |
| 36 | R-rostral middle frontal | -41 | -29 | 25 | 13 |
| 37 | R-superior frontal | -7 | 0 | 56 | 174 |
| 38 | R-superior parietal | -23 | 76 | 40 | 1427 |
| 39 | R-insula | -35 | -10 | 6 | 13 |
| 40 | L-cerebellar cortex | 34 | 55 | -31 | 971 |
| 41 | L-dorsal thalamus | 14 | 22 | 3 | 110 |
| 42 | L-caudate | 15 | 7 | 17 | 35 |
| 43 | L-putamen | 28 | 4 | 3 | 40 |
| 44 | L-pallidum | 18 | 7 | -3 | 22 |
| 45 | L-hippocampus | 24 | 24 | -15 | 35 |
| 46 | L-amygdala | 22 | 2 | -20 | 12 |
| 47 | L-ventral DC | 21 | 20 | -10 | 53 |
| 48 | R-cerebellar cortex | -28 | 64 | -30 | 1276 |
| 49 | R-dorsal thalamus | -15 | 25 | 0 | 51 |
| 50 | R-caudate | -16 | -1 | 14 | 16 |
| 51 | R-putamen | -26 | 1 | 2 | 37 |
| 52 | R-hippocampus | -28 | 14 | -19 | 53 |
| 53 | R-ventral DC | -15 | 16 | -13 | 44 |
|  |  |  |  |  |  |

**B. Task-negative ROIs**

| **No.** | **Label** | **MNI coordinate**  **X Y Z** | | | **Number of voxels** |  |
| --- | --- | --- | --- | --- | --- | --- |
| 1 | L-caudal anterior cingulate | 2 | -24 | 24 | 37 | |
| 2 | L-caudal middle frontal | 39 | -14 | 48 | 18 | |
| 3 | L-cuneus | 2 | 87 | 17 | 81 | |
| 4 | L-inferior parietal | 51 | 70 | 22 | 250 | |
| 5 | L-isthmuscingulate | 4 | 50 | 20 | 148 | |
| 6 | L-lateral orbitofrontal | 27 | -24 | -13 | 175 | |
| 7 | L-lingual | 3 | 70 | -10 | 40 | |
| 8 | L-medial orbitofrontal | 4 | -47 | -3 | 576 | |
| 9 | L-middle temporal | 60 | 16 | -16 | 191 | |
| 10 | L-paracentral | 5 | 37 | 45 | 176 | |
| 11 | L-pars opercularis | 61 | -11 | 20 | 21 | |
| 12 | L-postcentral | 54 | 28 | 35 | 402 | |
| 13 | L-posterior cingulate | 5 | 32 | 33 | 244 | |
| 14 | L-precentral | 48 | 11 | 23 | 262 | |
| 15 | L-precuneus | 5 | 65 | 27 | 1201 | |
| 16 | L-rostral anterior cingulate | 3 | -37 | 6 | 518 | |
| 17 | L-rostral middlefrontal | 27 | -47 | 27 | 824 | |
| 18 | L-superior frontal | 6 | -46 | 27 | 1703 | |
| 19 | L-superior parietal | 12 | 81 | 34 | 325 | |
| 20 | L-superior temporal | 56 | 13 | -3 | 424 | |
| 21 | L-supramarginal | 63 | 39 | 22 | 1071 | |
| 22 | L-transverse temporal | 45 | 22 | 5 | 62 | |
| 23 | L-insula | 39 | 3 | -4 | 243 | |
| 24 | R-bankssts | -54 | 41 | -3 | 70 | |
| 25 | R-caudal anterior cingulate | -2 | -26 | 24 | 48 | |
| 26 | R-caudal middle frontal | -37 | -13 | 49 | 289 | |
| 27 | R-cuneus | -3 | 88 | 14 | 94 | |
| 28 | R-inferior parietal | -49 | 60 | 16 | 269 | |
| 29 | R-inferior temporal | -55 | 10 | -32 | 20 | |
| 30 | R-isthmuscingulate | -3 | 49 | 18 | 193 | |
| 31 | R-lateral orbitofrontal | -29 | -27 | -13 | 368 | |
| 32 | R-lingual | -5 | 68 | -11 | 70 | |
| 33 | R-medial orbitofrontal | -4 | -46 | -4 | 945 | |
| 34 | R-middle temporal | -58 | 13 | -17 | 606 | |
| 35 | R-paracentral | -3 | 38 | 46 | 231 | |
| 36 | R-pars opercularis | -53 | -12 | 10 | 340 | |
| 37 | R-pars orbitalis | -48 | -33 | -8 | 172 | |
| 38 | R-pars triangularis | -48 | -22 | 2 | 185 | |
| 39 | R-postcentral | -47 | 22 | 19 | 144 | |
| 40 | R-posterior cingulate | -3 | 30 | 34 | 312 | |
| 41 | R-precentral | -50 | 3 | 14 | 162 | |
| 42 | R-precuneus | -5 | 63 | 27 | 1060 | |
| 43 | R-rostral anterior cingulate | -4 | -35 | 8 | 394 | |
| 44 | R-rostral middle frontal | -28 | -50 | 22 | 1637 | |
| 45 | R-superior frontal | -8 | -41 | 35 | 3199 | |
| 46 | R-superior parietal | -13 | 74 | 44 | 254 | |
| 47 | R-superior temporal | -52 | 3 | -9 | 884 | |
| 48 | R-supramarginal | -53 | 31 | 17 | 278 | |
| 49 | R-frontal pole | -5 | -63 | 1 | 80 | |
| 50 | R-temporal pole | -33 | -20 | -26 | 18 | |
| 51 | R-insula | -39 | 0 | -5 | 285 | |
| 52 | L-cerebellar cortex | 7 | 51 | -21 | 60 | |
| 53 | L-dorsal thalamus | 7 | 24 | 1 | 51 | |
| 54 | L-caudate | 11 | -11 | 6 | 98 | |
| 55 | L-putamen | 23 | -6 | -4 | 74 | |
| 56 | L-hippocampus | 26 | 24 | -19 | 19 | |
| 57 | L-accumbens | 7 | -9 | -8 | 54 | |
| 58 | L-ventral DC | 9 | 13 | -12 | 95 | |
| 59 | R-cerebellar cortex | -8 | 48 | -18 | 75 | |
| 60 | R-dorsal thalamus | -9 | 21 | 3 | 72 | |
| 61 | R-caudate | -10 | -11 | 8 | 64 | |
| 62 | R-putamen | -19 | -7 | -5 | 125 | |
| 63 | R-hippocampus | -23 | 17 | -19 | 23 | |
| 64 | R-accumbens | -10 | -11 | -6 | 25 | |
| 65 | R-ventral DC | -8 | 13 | -11 | 85 | |
|  |  |  |  |  |  | |
